# EFFECT OF SELF-GUIDED INTERVENTIONS ON MENTAL HEALTH OF INFORMAL DEMENTIA CAREGIVERS: A SYSTEMATIC REVIEW

**DOI:** 10.1093/geroni/igad104.2219

**Published:** 2023-12-21

**Authors:** Eunjung Ko, Thanchanok Wongvibul, Karen Rose, Jin Jun

**Affiliations:** The Ohio State University, Columbus, Ohio, United States; The Ohio State University, Columbus, Ohio, United States; The Ohio State University, Columbus, Ohio, United States; The Ohio State University, Columbus, Ohio, United States

## Abstract

Informal caregivers of people living with dementia often experience emotional and mental distress due to the demands of caregiving. Self-guided interventions aimed at improving the mental health of these caregivers have emerged, but their effectiveness remains understudied. We systematically examined the effects of self-guided interventions on stress, burden, and mental health of informal caregivers of people living with dementia. PubMed, CINAHL, PsycINFO, Scopus, and Embase databases were searched using relevant search terms for the study aims. Included articles focused on informal caregivers of people living with dementia and self-guided interventions to improve psychological or mental health. Sixteen articles and 1,182 samples were included in this review. Self-guided interventions covered topics on caregiving skills, emotional self-care, and/or information on social or financial resources related to caregiving. Outcomes of each study included stress, burden, depressive symptoms, anxiety, distress, quality of life, self-efficacy, positive aspects of caregiving, and loneliness. Depressive symptoms and burden were the most frequently measured outcomes. Stress was generally reduced after the interventions, whereas the results of burden and mental health from each intervention were inconsistent. Interventions lasting shorter than three months were more likely to be effective compared to longer interventions. Self-guided interventions may be useful for improving mental health of informal caregivers of people living with dementia due to its low time burden, ease-to-access, and affordability. Future research is needed to determine the optimal length and components of self-guided interventions and to collaborate with clinicians for wider distribution to informal caregivers of people living with dementia.

